# Objective Assessment of Perioperative Anxiety using Functional Near-infrared Spectroscopy in Elderly Patients: A Prospective Randomized Observational Pilot Study

**DOI:** 10.7150/ijms.89287

**Published:** 2023-10-24

**Authors:** Hyae Jin Kim, Dowon Lee, Hyun-Su Ri, JongKwan Choi, Jinhyung Choi, Seung Joon Rhee, Jiseok Baik, Boo-young Hwang, Gayoung Park, Jihyun Cha, Sang Don Lee

**Affiliations:** 1 Department of Anesthesia and Pain Medicine, School of Medicine, Pusan National University, Busan, South Korea.; 2 Department of Anesthesia and Pain Medicine, Biomedical Research Institute, Pusan National University Hospital, Pusan, South Korea.; 3 Department of Anesthesiology and Pain Medicine, School of Medicine, Kyungpook National University, Daegu, South Korea.; 4 OBELAB Inc, Seoul, South Korea.; 5 Department of Orthopedic Surgery, Biomedical Research Institute, Pusan National University Hospital, Pusan National University School of Medicine, Busan, South Korea.; 6 Department of Urology, Pusan National University Yangsan Hospital, Pusan National University School of Medicine, Yangsan, South Korea.

**Keywords:** Anesthesia, Spinal, Anxiety, Perioperative period, Spectroscopy, Near-Infrared.

## Abstract

**Background:** Assessing and managing patient anxiety is essential to reduce postoperative complications in elderly patients. However, monitoring patient anxiety objectively is impossible. This study aimed to investigate the correlation between the level of fNIRS signals and anxiety in patients aged 65 and older undergoing artificial joint replacement surgery.

**Material and Methods:** Sixty patients aged ≥65 years scheduled for elective total knee arthroplasty under spinal anesthesia were included. To differentiate the degree of anxiety, the patients were randomly divided into three groups, each consisting of 20 patients (group 1: administered normal saline as a placebo; groups 2 and 3: administered dexmedetomidine at a rate of 0.2 and 0.5 μg/kg/h, respectively, for 10 min). Functional near-infrared spectroscopy was measured continuously for 10 min in each session (session 1: pre-anesthetic period; session 2: immediately after the spinal anesthesia period; session 3: normal saline or dexmedetomidine receiving period) in all patients. Vital signs were measured thrice at 5-min intervals during each session. State-Trait Anxiety Inventory -S (STAI-S) and Ramsay Sedation Scale (RSS) scores were assessed at the end of each session.

**Results:** The STAI-S score was significantly correlated with power of bandwidth (*p* = 0.034). In addition, the RSS score was significantly correlated with BW 1, 2, and 3 (*p* = 0.010, *p* < 0.001, and *p* = 0.003, respectively).

**Conclusion:** The STAI-S score and BW 3 were significantly correlated, suggesting that fNIRS might help objectively and directly monitor anxiety levels.

## Introduction

Anxiety, an emotional state that patients commonly experience, occurs in 60%-80% of patients undergoing surgery 1. It can cause physical and emotional problems, and increased anxiety can negatively affect physiological responses, such as hypertension and arrhythmias during anesthesia 2, 3. Anxiety during the perioperative period is associated with various postoperative complications, particularly postoperative delirium (POD), in elderly patients 4-7. POD in the elderly is associated with various adverse health effects, including cognitive and functional decline, high morbidity and mortality, increased intensive care unit and hospital stays, and increased risk of institutionalization, resulting in additional medical costs 8.

Therefore, evaluating and managing anxiety levels perioperatively is essential to reduce postoperative complications, including POD, in the elderly patients. Although managing anxiety through various drugs, including anesthetics, is possible, objective and direct monitoring of the degree of anxiety experienced by patients is impossible. While subjective assessment can provide valuable insights, anxiety is a complex condition, and relying solely on a patient's subjective experience may not provide a comprehensive understanding of all aspects of the condition. Therefore, objective assessment is essential for more effective understanding of anxiety and optimizing its treatment 9.

Dexmedetomidine is highly selective alpha-2 adrenergic agonists with sedative, analgesic, anxiolytic, sympatholytic and minimal respiratory depression properties 10-12. Dexmedetomidine has been primarily used as a sedative and anesthetic adjunct in various medical settings, including during spinal anesthesia. By adding dexmedetomidine to the spinal anesthesia regimen, patients may experience enhanced sedation, leading to increased comfort and relieve anxiety during the procedure. Dexmedetomidine has various cerebral effect and the drug itself can influence cerebral blood flow depending on drug concentration and cerebral tissue oxygenation, it could potentially impact fNIRS signal as well 13-16. To investigate effects of anxiety on fNIRS signal, the dosage of dexmedetomidine should be lowered to minimize its own cerebral effects.

Functional near-infrared spectroscopy (fNIRS) is a noninvasive continuous monitoring technique that allows to measure changes in cerebral tissue oxygen saturation by detecting changes in oxyhemoglobin (ΔHbO_2_) and deoxyhemoglobin (ΔHbR) concentrations in the cerebral cortex. In addition, dynamic changes in oxyhemoglobin and deoxyhemoglobin levels can be measured through multiple channels 17. NIRSIT ON (OBELAB Inc., Seoul, Republic of Korea) is a device to measure regional oxygen saturation and hemodynamic variation (ΔHbO_2,_ ΔHbR) using fNIRS technique in the prefrontal cerebral cortex by placing two patches. In addition to measuring changes in hemoglobin concentration, near-infrared light can penetrate several centimeters into tissue, so that the spontaneous oscillations of oxyhemoglobin (HbO_2_) and deoxyhemoglobin (HbR) in brain parenchymal tissue can be measured. The spontaneous oscillation signals can be categorized in to four frequency bandwidths (BW) according to their specific physiological origins; Very low-frequency oscillations (VLFO) (0.01-0.04 Hz) are associated with neurogenic; low-frequency oscillations (LFO) (0.04-0.15 Hz) are related to myogenic activation; a frequency of 0.16-0.6 Hz is affected by respiration; and a frequency of 0.8-1.5 Hz is affected by heartbeat 18-22. These spontaneous oscillations are observed with fNIRS, and can be analyzed in the frequency domain as a power spectral density (PSD). Power spectral density is a tool used to analyze the frequency components of a signal and characterizes the frequency distribution of signal variance in a time series. In fNIRS signals, PSD shows how much signal power is present within different frequency ranges and allows to extract information mainly related to brain activity 23. Spontaneous oscillations can be distinguished by spectral analysis. Spectral analysis of oxygenation signals has been introduced as a method for the evaluation of regulatory mechanisms of cerebral and tissue vascular system 19, 24.

Perioperative anxiety can cause physiological changes, including those in the sympathetic nervous system and patient respiration 25, 26. Therefore, these changes may have affected the power of previously mentioned specific frequency bandwidths in fNIRS 27, 28. Considering that fNIRS reflects cortical hemodynamic changes and that PSD provides information of a specific physiological origin, this study aimed to investigate the correlation between the level of fNIRS signals and anxiety in patients aged 65 and older undergoing total knee arthroplasty (TKA) surgery.

## Material and Methods

### Ethical Statement

This prospective, randomized observational study was conducted at the Pusan National University Hospital. The study was approved by the Institutional Review Board of the Pusan National University Hospital (IRB number: D-1910-027-083, Busan, South Korea) and was registered with the Clinical Research Information Service (KCT0007692, http://cris.nih.go.kr). Patients were informed about all the possible adverse effects and told they could stop whenever they wanted. All patients signed an informed consent form prior to the surgery.

### Patients

**Figure 1:**
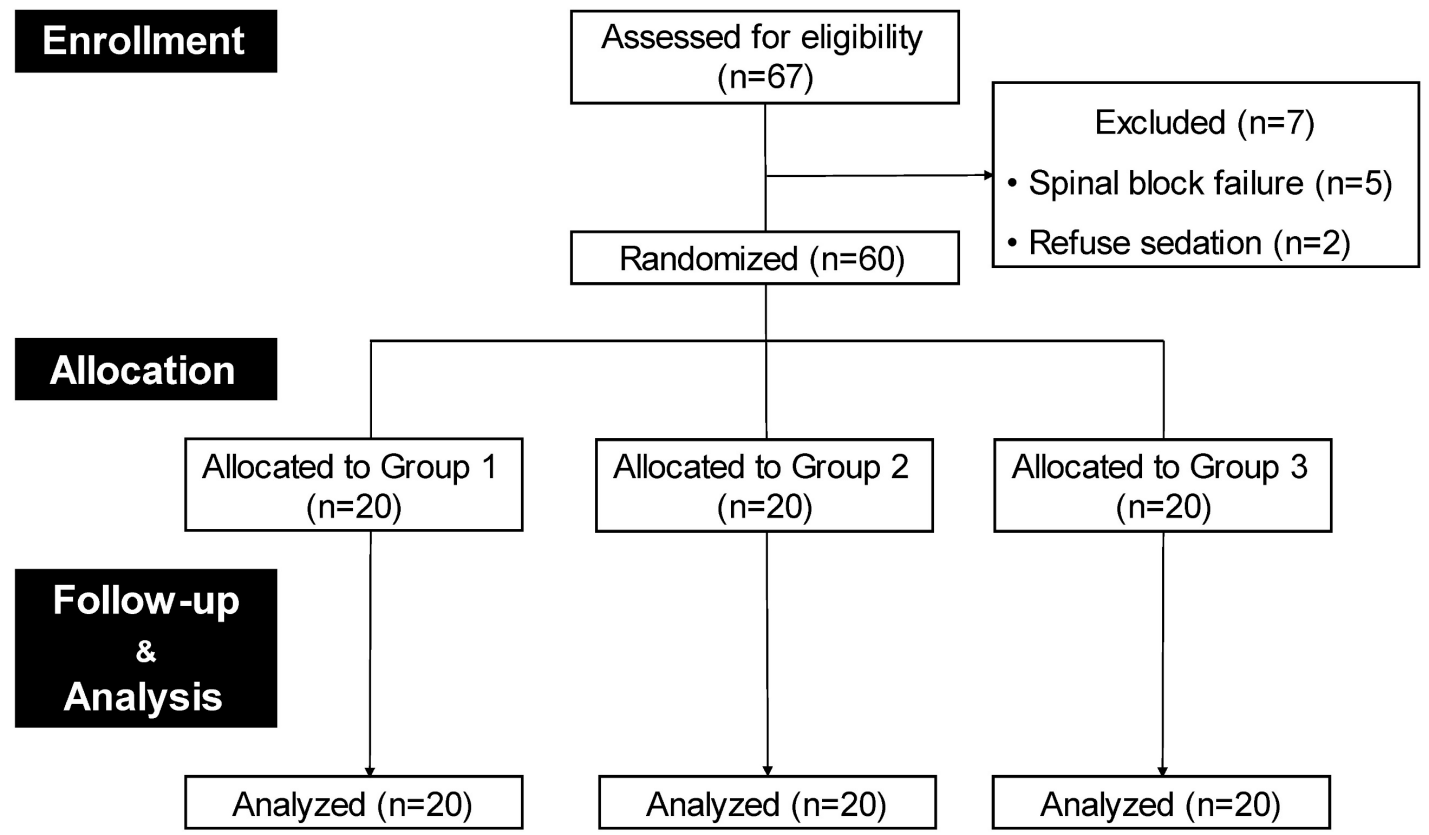
** Study flow diagram.** Design of the study to evaluate the correlation of anxiety with fNIRS signals. Finally, 60 patients were included in this study. They were randomly assigned to one of the three groups (20 per group) with different anxiety levels using different dexmedetomidine dosage. Group1, a placebo group, received normal saline at a rate of 5 mL/h for 10 min at the start of surgery Groups 2 and 3 received dexmedetomidine 0.2 and 0.5 μg/kg/h, respectively, for over 10 min at the beginning of the surgery.

Patients with American Society of Anesthesiologists (ASA) physical status I or II aged ≥65 years who were scheduled for elective total knee arthroplasty (TKA) under spinal anesthesia were eligible. The exclusion criteria were as follows: contraindications to spinal anesthesia; refusal of sedation during surgery; previously diagnosed psychiatric disorders, cranial nerve disorders, or cognitive impairment; and chronic administration of sedatives or opioids. Sixty-seven patients were initially screened, and 60 were enrolled in this pilot study. Figure 1 illustrates the study flow diagram.

### Grouping

The primary endpoint of this study was to determine different anxiety levels and identify the corresponding frequency bands. The purpose of dividing the patients into three groups was not for inter-group comparisons but rather to vary the levels of anxiety. Therefore, 60 patients were randomly assigned to one of the three study groups (20 patients per group) with different anxiety levels. Group 1 (placebo group) received normal saline at a rate of 5 mL/h for 10 min at the start of surgery. Groups 2 and 3 received dexmedetomidine for over 10 min at the beginning of the surgery. Dexmedetomidine was diluted with 0.9% saline at a 4-μg/mL concentration. Groups 2 and 3 were administered dexmedetomidine at a dose of 0.2 and 0.5 μg/kg/h, respectively. Computer-generated permuted-block randomization was used with a block size of three and an allocation ratio of 1:1:1. The anesthesiologist could not be blinded because he was in charge of anesthetic care; a blinded investigator, who was not directly involved in anesthetic care, collected all data.

### Spinal anesthesia and management

All patients received routine preoperative care without premedication. On arrival in the operating room, standard monitoring was initiated, including noninvasive automated blood pressure, electrocardiography, and pulse oximetry. Spinal anesthesia was induced in the lateral decubitus position using a 25-gauge Quincke spinal needle and 10-12 mg of hyperbaric 0.5% bupivacaine in the L3/L4 or L4/L5 intervertebral space. The level of sensory block was assessed using cold sensitivity to alcohol swabs 10 min after intrathecal injection. After achieving an adequate sensory block (at the T10 level), the patients were prepared for surgery, and oxygen was administered at 3 L/min via a nasal cannula. Spinal anesthesia-induced hypotension was defined as a systolic BP (SBP) < 90 mmHg and a decrease in baseline SBP > 20% after the intrathecal injection. The patient was initially treated with 200 mL of a balanced crystalloid solution when hypotension occurred. When hypotension persisted for 3 min, 5 mg of ephedrine was administered intravenously and repeated every 3 min, depending on the subsequent improvement. Patients with incomplete or high-level spinal anesthesia (sensory block level > T4) and low blood pressure (systolic blood pressure < 90 mmHg) that persisted despite appropriate treatment were excluded from the study.

### Measurement of anxiety and sedation

The state and trait anxiety inventory (STAI) scores were used to assess anxiety during this study 29, 30. The STAI consists of two types of scoring system, the STAI-S (state anxiety) and the STAI-T (trait anxiety). The STAI-T scale measures a person's general, stable level of anxiety over time, the STAI-S scale asses a person's current, temporary feeling of anxiety or tension. In this experiment, the STAI-S was used to measure anxiety specific to the current moment or specific situation (Table 1). Reversed scoring items are 1, 2, 5, 8, 10, 11, 15, 16, 19, and 20. Higher scores indicate higher levels of anxiety. The Ramsay Sedation Scale (RSS) was used to evaluate the degree of sedation (Table 2). The RSS consists of six levels, each describing a different level of sedation. The RSS was assessed by an investigator blind to the assigned groups, at the end of each session.

### Functional near-infrared spectroscopy (fNIRS) measurement using NIRSIT ON

Functional NIRS (fNIRS) was measured continuously for 10 min in each session using NIRSIT ON (OBELAB Inc., Seoul, Republic of Korea) that can measure cerebral oxygen saturation (rSO_2_) and hemodynamic signals, such as oxyhemoglobin (HbO_2_) and deoxyhemoglobin (HbR). The fNIRS system measures changes in hemodynamics employing lasers with wavelengths of 780 nm and 850 nm to measure the oscillation of the oxyhemoglobin and deoxyhemoglobin concentration change with higher temporal resolution. Optical density change for each wavelength from the cerebral tissue was sampled at a frequency of 8.138 Hz, and the hemodynamic change (ΔHbO_2_) was calculated based on modified-Beer Lambert's law 31. fNIRS has higher spatial resolution compared to conventional NIRS, used for measuring the cortical hemodynamic responses and functional connectivity.

**Figure 2:**
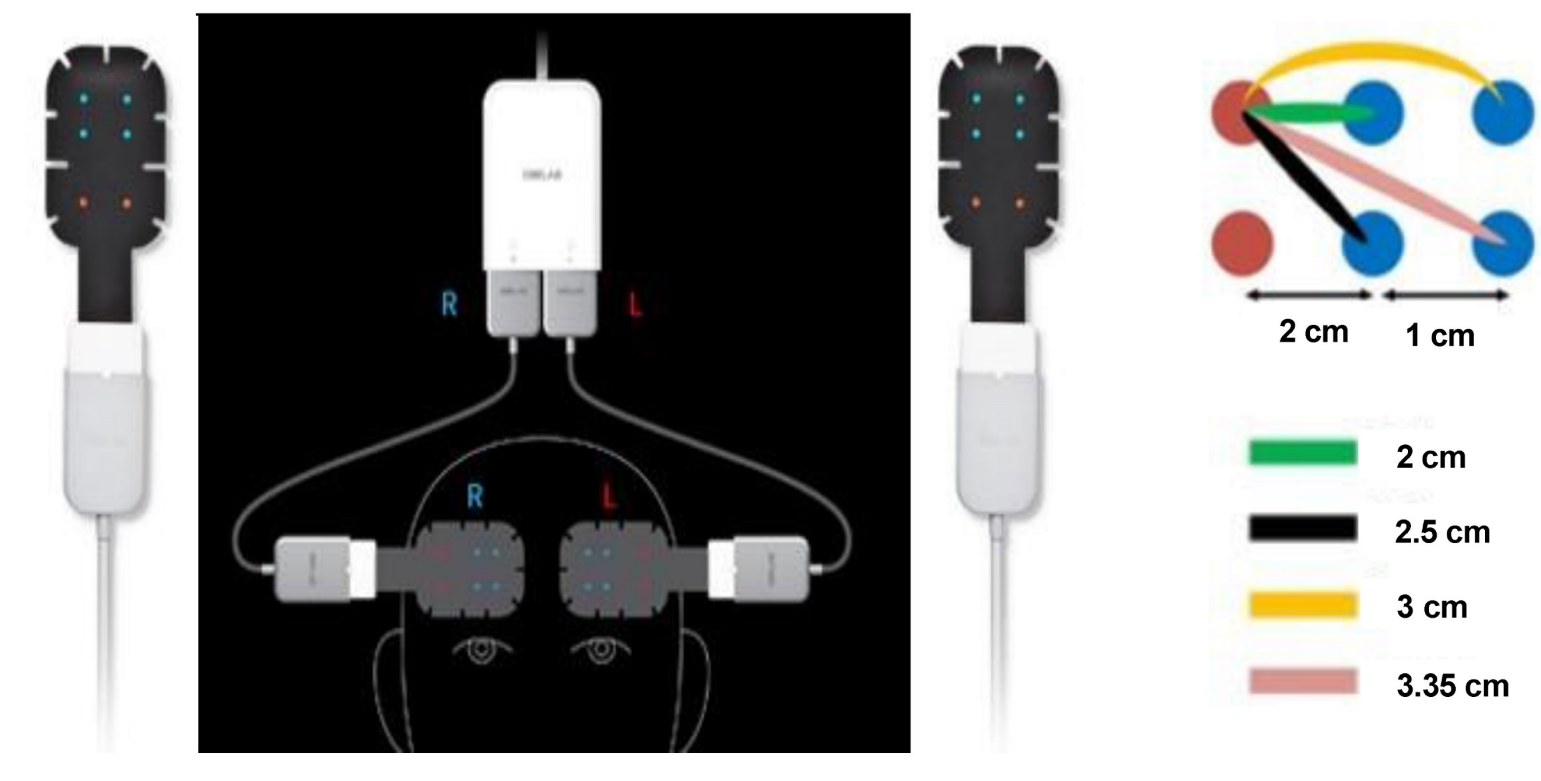
** NIRSIT-ON Patch.** Two patches (left and right) (Figure 2A) were operated independently to measure the HbO_2_ of the brain. Each patch has two channels (Figure 2B): short (2 and 2.5 cm, green and black lines) and long (3 and 3.35 cm, yellow and pink lines).

The NIRSIT ON sensor is independently composed of two patches (Figure 2) which measures ΔHbO_2_ in the cerebral cortex 32, 33. Two sensors should be placed on the patients' forehead, with one positioned above the right cerebral hemisphere and the other above the left cerebral hemisphere. One adhesive sensor patch was attached above the right eyebrow, while the second one above the left eyebrow. HbO_2_ calculated from the two channels that 3-cm separated source and detector pairs in each patch was averaged and used. To check the variability of the hemodynamic information, the signals were filtered using a passband of 0.005- 3 Hz. There are both short and long channels as shown in figure 2. Only the long channels were used to assess cortical activity more precisely, preprocessing for the short channel was not applicable.

The power of each bandwidth (BW) was calculated within frequencies between 0.01 and 1.5 Hz and divided into four non-overlapping BW channels. BW 1: 0.01-0.04 Hz (neurogenic activity), BW 2: 0.04-0.15 Hz (myogenic activity), BW 3: 0.16-0.6 Hz (respiration), and BW 4: 0.8-1.5 Hz (heartbeat).

### Outcome variables

**Figure 3:**
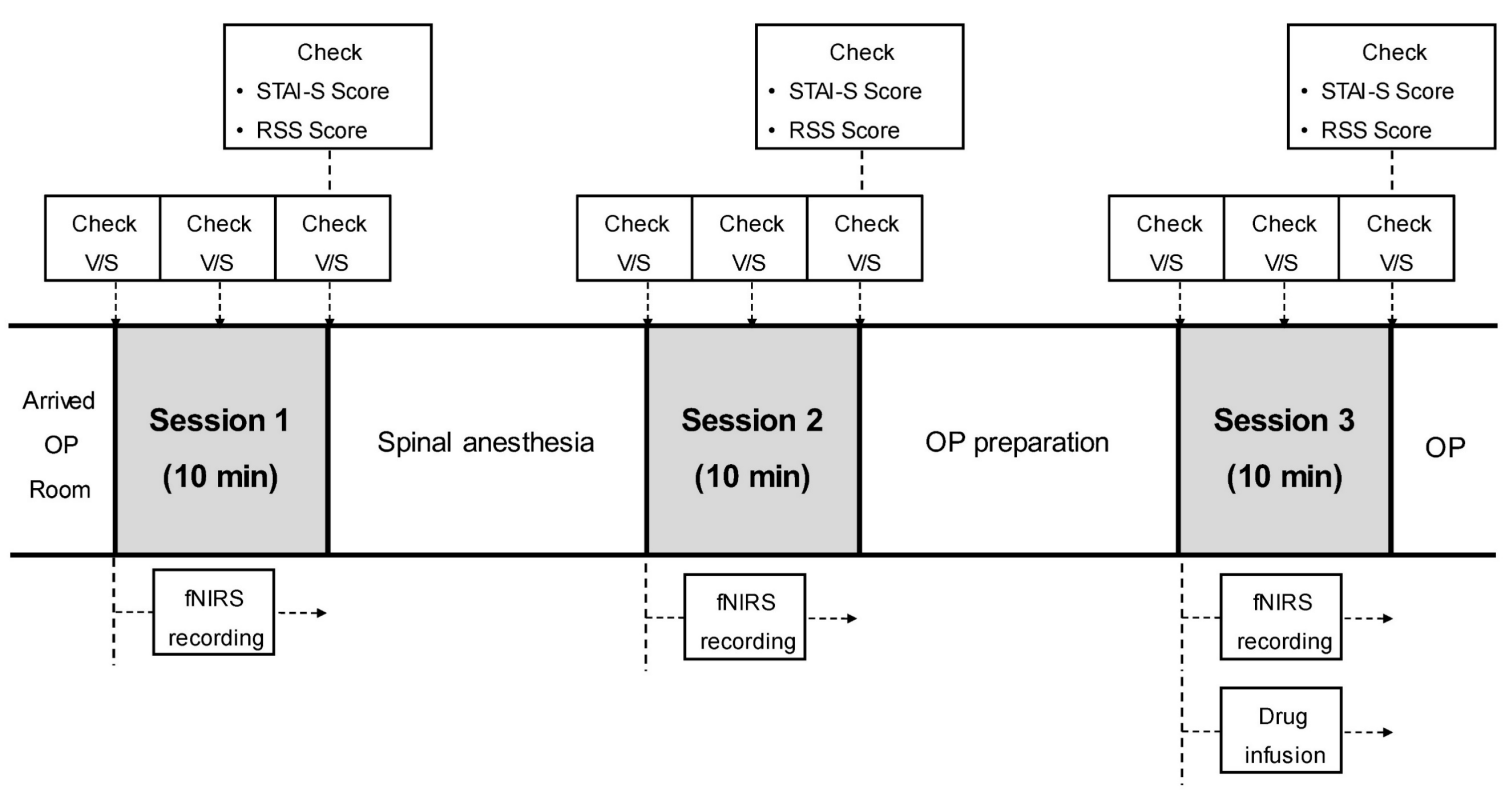
** Clinical timeline of the study protocol.** Vital signs, STAI-S, RSS, and fNIRS were recorded thrice: pre-anesthesia (session 1), after spinal anesthesia (session 2), and 10 min after drug infusion (session 3). Vital signs were checked thrice in each session at 5-min intervals. *STAI-S, state-trait anxiety inventory; RSS, Ramsay sedation scale; V/S, vital signs; fNIRS, functional near-infrared spectroscopy.

All variables were measured repeatedly over three sessions (session 1: pre-anesthetic period, session 2: immediately after the spinal anesthesia period, and session 3: normal saline or dexmedetomidine administration period) for 10 min each. At each session, vital signs (mean blood pressure (MBP), heart rate (HR), and oxygen saturation (SpO_2_)) were measured and recorded thrice at 5-min intervals. The State-Trait Anxiety Inventory (STAI)-S and Ramsay Sedation Scale (RSS) scores were assessed at the end of the session, and fNIRS consecutive records were automatically stored throughout the sessions (Figure 3).

### Statistical analysis

Statistical analyses were performed using SPSS version 24 (IBM Corp., Armonk, NY, USA), R 34 and R-studio version 1.2 35. Normally distributed data are expressed as mean ± standard deviation, and non-normally distributed data are expressed as median. All variables were tested for normality using the Shapiro-Wilk test. The variables BW 3 and BW 4 were converted using Box-Cox transformation because they did not satisfy the normal distribution. To assess changes in the clinical indicators STAI-S, RSS, HR, MBP, and SpO_2_ over the course of the session and the session with group interaction, we used repeated-measures ANOVAs and checked for sphericity. Additionally, the correlations between the power spectral density values at the four frequency intervals (BW) and all variables were assessed through a repeated measurement correlation analysis using linear mixed-effects models. All *p*-values were two-sided, and a *p*-value < 0.05 was considered to indicate statistical significance.

## Results

A total of 67 patients scheduled for TKA were assessed for eligibility, and seven were excluded (five due to spinal block failure and two for refusal of sedation). The remaining 60 patients were assigned to one of the three groups and completed the study (Figure 1).

Table 3 shows the patient characteristics, including age, sex, height, weight, and ASA classification. MBP, HR, and SpO_2_ were measured nine times, and the STAI-S and RSS scores were assessed thrice. Table 3 shows the mean MBP, HR, and SpO_2_ values in the first session and the initially assessed STAI-S and RSS scores. To confirm that there were no differences in the baseline vital signs before dexmedetomidine administration in all patients, MBP, HR, and SpO_2_ were measured and compared during the first session.

**Figure 4:**
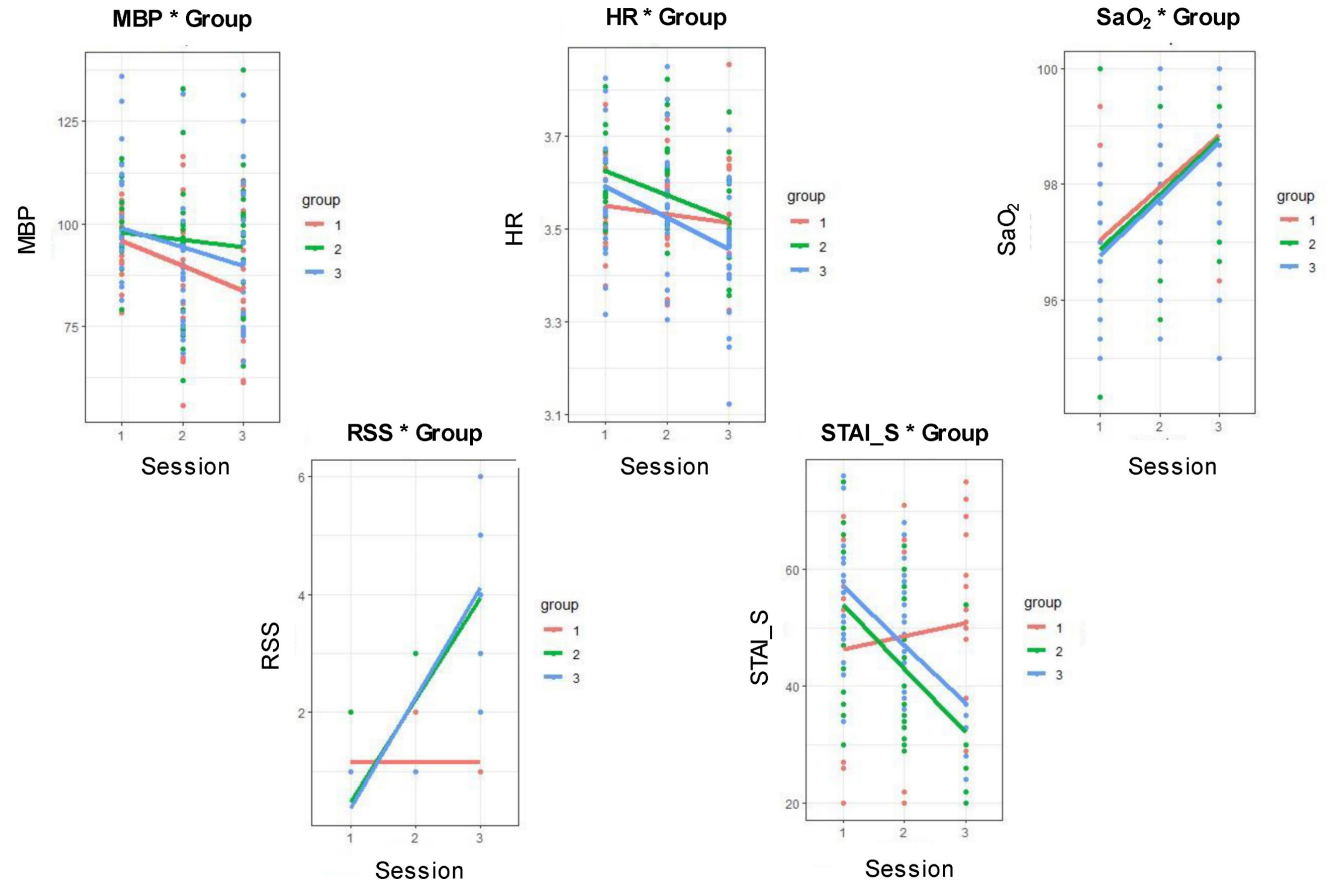
** Variables in each session and group.** MBP and SpO_2_ differed significantly between sessions (*p* = 0.039 and *p* = 0.001, respectively). The RSS and STAI-S scores differed significantly among groups, sessions, and groups with sessions (*p* < 0.001, *p* = 0.006, and *p* < 0.001, respectively; *p* < 0.001, *p* = 0.001, and *p* < 0.001, respectively). MBP, mean blood pressure; HR, heart rate; SpO_2_, oxygen saturation; RSS, Ramsay Sedation Scale; STAI-S, State-Trait Anxiety Inventory.

Patient characteristics and the variables assessed in the first session did not differ significantly among the three groups. Figure 4 shows the variables assessed as the sessions progressed in each group. MBP and SpO_2_ differed significantly between the sessions (*p* = 0.039 and *p* = 0.001, respectively). However, the RSS and STAI-S scores were significantly different among groups, sessions, and groups with sessions (RSS: *p* < 0.001, *p* = 0.006, and *p* < 0.001; STAI-S: *p* < 0.001, *p* = 0.001, and *p* < 0.001). These results demonstrated that the study design was appropriate for implementing differentiated anxiety levels in each group and session.

**Figure 5:**
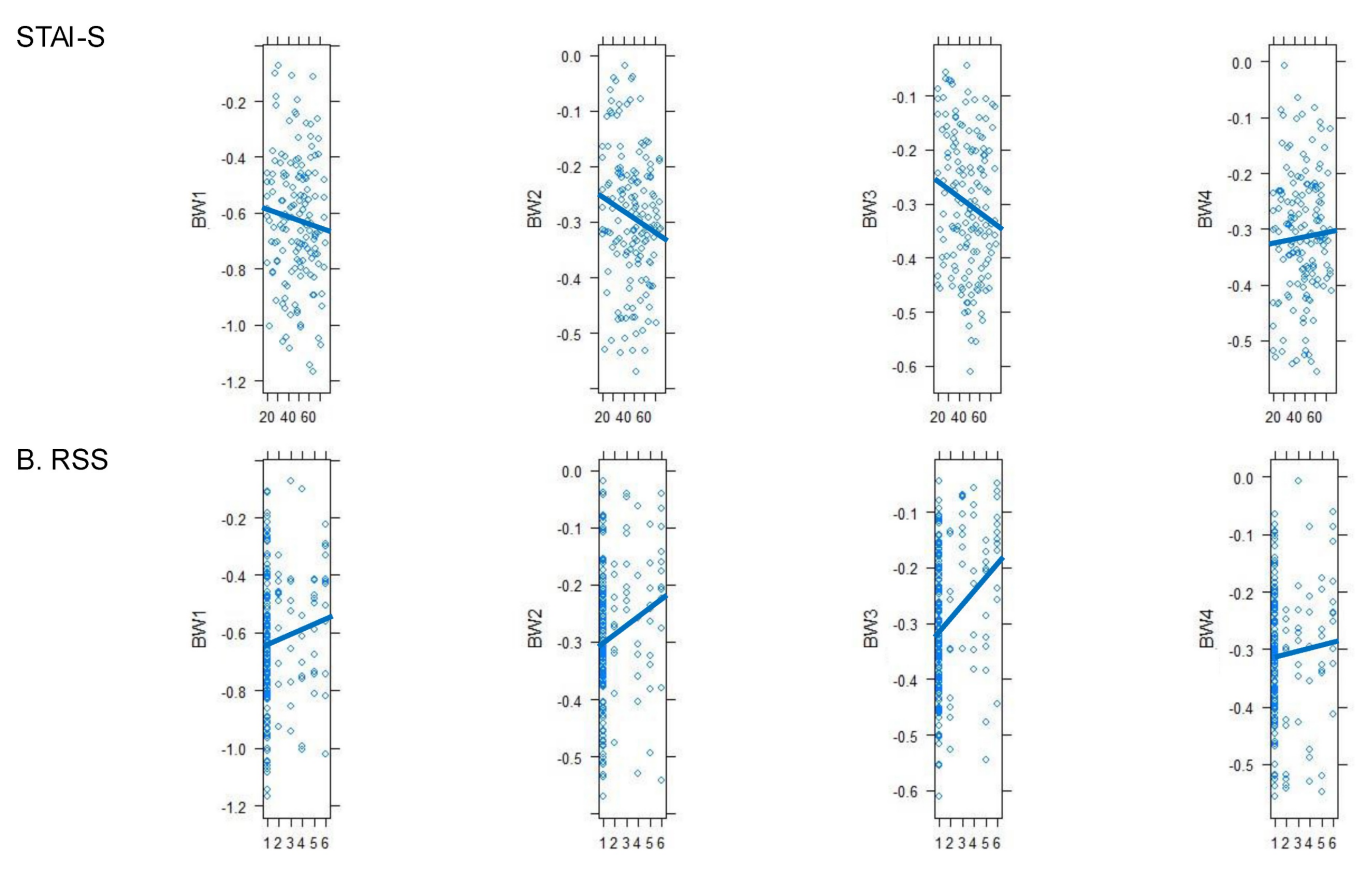
** STAI-S and RSS.** The STAI-S score significantly correlated with BW 3 (*p* = 0.034), and a tendency was also observed with BW 2 (*p* = 0.061; Figure 5A). The RSS score was significantly correlated with BW 1, 2, and 3 (*p* = 0.010, *p* < 0.001, and *p* = 0.003, respectively; Figure 5B). Other variables and BW were positively correlated. *RSS, Ramsay Sedation Scale; STAI-S, State-Trait Anxiety Inventory; BW, bandwidth.

The primary outcome was the identification of power of BW at four frequency intervals (BW 1-4) directly related to anxiety (STAI-S score), rather than comparing groups and sessions. All data were first merged for descriptive statistics: 60 patients, three sessions per patient, for a total of 180 data points (Table 4). The STAI-S score significantly correlated with BW 3 (*p* = 0.034), and a tendency was also observed with BW 2 (*p* = 0.061; Figure 5A). In addition, the RSS score was significantly correlated with BW 1, 2, and 3 (*p* = 0.010, *p* < 0.001, and *p* = 0.003, respectively; Figure 5B), and the other variables were not correlated with BW.

## Discussion

This study demonstrated that the STAI-S score, which reflects anxiety levels, was significantly correlated with BW3 in the power of bandwidth analysis using fNIRS during the perioperative period in patients aged 65 and older undergoing total knee arthroplasty (TKA) surgery.

Anxiety is a pathological condition characterized by fear and physical symptoms due to hyperactivity of the autonomic nervous system 36. Perioperative anxiety has been associated with unfavorable complications, such as nausea, vomiting, cardiovascular disturbances, including tachycardia and hypertension, and an increased risk of infection 2, 37. Anxiety also increases the requirement of anesthetics to cause unconsciousness and may indirectly increase the risk of awareness 38. Pain is the most common complaint of perioperative anxiety and increased requirement for postoperative analgesia 39. Preoperative anxiety negatively affects surgical satisfaction and is related to surgical success rates and postoperative complications 40. A large proportion (60%-80%) of surgical patients experience substantial preoperative anxiety 1, 6, 41, 42. Perioperative anxiety can cause serious complications in the elderly patients. Mıngır et al. showed that anxiety scores on the STAI had a positive and statistically significant correlation with patient age 36. Severely anxious elderly patients have significant medical and cognitive complications, including a higher stroke risk and cognitive decline. Thus, it is crucial to decipher the effects of anxiety on the aging brain to develop appropriate interventions and prevent further decline.

The dexmedetomidine dose used to determine the degree of anxiety differed among the groups. Usually, the maintenance dose of dexmedetomidine in the intensive care unit (ICU) for adults is approximately 0.2-0.7 μg/kg/h. To induce light sedation, dexmedetomidine was not administered over 0.7 μg/kg/h in this study. Therefore, patients were divided into groups 2 (administered dexmedetomidine at a rate of 0.2 μg/kg/h) and 3 (administered dexmedetomidine at a rate of 0.5 μg/kg/h). Dexmedetomidine exhibits various contradictory effects on cerebral physiology. Numerous *in vitro* and *in vivo* investigations have demonstrated that dexmedetomidine possesses neuroprotective effect during ischemic conditions 11, 16, 43. Neuroprotective properties of dexmedetomidine make it a suitable agent for neurosurgery. Existing evidence supports a dose-dependent decline in both global and regional cerebral blood flow (CBF) via direct vasoconstriction of the cerebral vasculature and indirectly via effects on the intrinsic neural pathways 13, 14, 44. In particular, Gatto et al. have studied the impact of dexmedetomidine on cerebral tissue oxygenation using near-infrared spectroscopy, and found a decrease in oxyhemoglobin during dexmedetomidine injection 14. From these findings, it can be inferred that dexmedetomidine may have an impact on cerebral blood flow and fNIRS in its own drug effect. However, considering that there were no significant association in cerebral tissue oxygen saturation in the present study and the dosage we used was relatively low compared to usual sedation, the impact of dexmedetomidine on brain physiology may not have been substantial.

This study aimed to objectively determine how anxiety in elderly patients should be monitored. Accurately measuring preoperative anxiety levels is challenging. Several validated questionnaires are available and used to measure preoperative anxiety. These include the Amsterdam Preoperative Anxiety Information Scale, STAI, Hospital Anxiety and Depression Scale, Visual Analog Scale, and Multiple Affect Adjective Check List. Furthermore, it can be estimated indirectly by measuring the blood pressure, pulse rate, decreased HR variability, and patient irritability. It can also be estimated by measuring the plasma cortisol and urinary catecholamine levels 37. Objective assessment of anxiety is considered significantly superior to subjective evaluation in several aspects. Objective assessment allows quick diagnosis (current state), less recall bias and minimize the impact of cultural and linguistic factors on emotion and symptom questionnaire 9.

STAI is a validated and widely used instrument for measuring patient anxiety 38, 45. Here, the STAI-S scores correlated well with the sessions, groups, and groups with sessions (Figure 4). Because the purpose of this study was to set various anxiety levels, the anxiety level was well differentiated according to the different dexmedetomidine doses and to evaluate the correlation between the STAI-S score and fNIRS parameters.

As shown in Figure 4, the MBP differed between sessions because dexmedetomidine was administered in session 2. The RSS was also significantly different among sessions, groups, and groups with sessions, as dexmedetomidine was administered from session 2 at different concentrations between groups. Because oxygen at 3 L/min was administered via a nasal cannula after spinal anesthesia (from session 2), SpO_2_ increased during sessions 2 and 3. Therefore, SpO_2_ differed significantly between the sessions.

Cerebral hemodynamic response refers to the changes in blood flow, blood volume, and oxygenation in the brain in response to neural activity. Wavelet of spontaneous oscillation analysis was used to investigate functional relationships among different cerebral cortical areas using fNIRS signals 48. Functional studies that use hemodynamic responses, such as functional magnetic resonance imaging and fNIRS, exist as physiological fluctuations 49. These fluctuations have a distinct frequency band (0.01-2 Hz) and a specific physiological origin. Lower frequencies may reflect neurogenic (BW 1, -0.01 to 0.04 Hz), myogenic (BW 2, 0.04-0.15 Hz), while higher frequencies result from respiration (BW 3, 0.16-0.6 Hz) and cardiac activity (BW 4, 0.8-1.5 Hz). These physiological signals are commonly treated as noise as they may lead to incorrect interpretations of fNIRS activation. However, anxiety can have a significant impact on the cerebral hemodynamic response via vasoconstriction, increased heart rate, hyperventilation, cortisol release, and neurotransmitter imbalance. Therefore, physiologic frequency oscillation (BW in the present study) could be related to cerebral hemodynamic response result from anxiety. Among these BW parameters, the origin of BW 2 was the myogenic component and that of BW 3 was associated with respiration. In particular, BW 3 showed the strongest correlation with STAI-S, a score indicating the degree of anxiety among all measures. BW 3 is a wavelet closely related to respiration. In a state of reduced anxiety, various hemodynamic signs may change; in particular, the change in respiration becomes more stable when the anxiety is less than that in a state of restlessness. Changes in state anxiety often accompany respiratory changes, affecting arterial CO_2_ concentrations and causing significant changes in cerebral blood flow (CBF) 51. As fNIRS, a blood oxygen level-dependent method, is mediated by changes in CBF caused by changes in blood oxygenation, factors affecting CBF independent of local brain activity should be considered. To remove any artifact or noise from the sources, there need preprocessing step in fNIRS data collecting. However, only long channels were used to detect precise cortical activity in the present study and the preprocessing for the short channel was not applied. Respiratory alterations resulting from anxiety or anti-anxiety might affect CBF and could significantly change BW 3 in this study. In addition, BW 2 originates from myogenic factors and is affected by the muscles of the blood vessels. Anxiety stimulates autonomic overactivity and increases cardiovascular reactivity and blood pressure 25. Therefore, depending on the state of increased or decreased anxiety, stimulation and relief of the sympathetic nerve occurs, and accordingly, constriction and relaxation of the blood vessels occurs. Here, the STAI-S score showed a tendency towards BW 2.

In addition to the anxiety score (STAI-S), the RSS score also showed a close correlation with the wavelets, BW 1, 2, and 3 (Figure 5B). This can be explained in two ways. First, as the brain works more actively, its metabolism increases; therefore, the HbO_2_ concentration increases and that of HbR decreases 52. Conversely, as sedation deepens, brain metabolism and oxygenated hemoglobin levels decrease, and reduced hemoglobin levels increase, which affects the fNIRS measurement. Second, in cases of sedation caused by dexmedetomidine, blood vessel relaxation and decreased heart and respiratory rates may be caused by a decrease in the sympathetic nervous system. This may cause changes in BW, similar to anxiety. BW4 is generated by oscillations origination from heartbeats, closely related to heart rate. In this study, hear rate (HR) was not significant difference among different sessions (figure 4). This is why BW4 had no association with the RSS score.

Many studies have demonstrated the relationship between fNIRS monitoring and patient's emotion including anxiety 53-58. They revealed correlation of emotional change (i.e. anxiety, mental stress, depression) with fNIRS signals. Various fNIRS variables were used to prove emotional monitoring including concentration of oxyhemoglobin (HbO_2_), deoxyhemoglobin (HbR), power of wavelet (bandwidth) and wavelet coherences. The previous studies were based on change of cerebral hemodynamics during emotional change and proposed that fNIRS is a suitable device for monitoring various emotional changes. However, there is a lack of studies using fNIRS as a tool for measuring anxiety in patients undergoing regional anesthesia. Jiala et al. reported that preoperative multimedia information could reduce intraoperative anxiety in patients receiving regional anesthesia 59, the STAI score was used as a tool to measure anxiety. Mıngır et al. measured anxiety in patients receiving spinal anesthesia and found advanced age, female gender, and low American Society of Anesthesiologists (ASA) class affect perioperative anxiety, which were measured using the STAI score 36. Therefore, there have been few studies that objectively measure anxiety by analyzing patients' physiological signals. Hence, this study indicates the possibility for fNIRS to be utilized by means of measuring intraoperative anxiety.

This study had some limitations. First, because this study was intended for elderly patients aged ≥65, the results cannot be generalized to all age groups. Second, this was a pilot study; therefore, the sample size was relatively small. Third, the patients were mainly women, and all sexes were not included. Fourthly, a limitation of this experiment is its inability to introduce a more diverse range of anxiety levels among the patients. While the three different dosages of dexmedetomidine were employed to induce differing degrees of anxiety, it would be advantageous in future studies to explore a wider array of medication dosages, allowing for greater variations in anxiety levels. In particular, it needs to consider that a patients' anxiety level can be influenced by personal experiences, especially prior experiences with spinal anesthesia, as well as any comorbid conditions they may have. Therefore, there is a need for multifaceted tools to assess patients' anxiety comprehensively. This approach could provide valuable insights by comparing fNIRS signals across different levels of anxiety. Finally, we failed to quantify the degree of anxiety using fNIRS, such as the bispectral index or entropy, but only found that BW and degree of anxiety are related. Therefore, extensive population studies are required to include various age groups and a balanced sex ratio develop a method to quantify the degree of anxiety measured by fNIRS.

In conclusion, power of bandwidth (BW) analysis proved to be an objective parameter that reflects the degree of anxiety using fNIRS. In particular, BW 3 (0.16-0.6 Hz) was a very reliable value related to the STAI-S score. A large population study is required to apply this result to various age groups and other sexes and quantify the level of anxiety in the future.

## Figures and Tables

**Table 1:** ** STAI-S questionnaire.** The state-trait anxiety inventory -state (STAI-S) consists of 20 questions that measure a patient's current state of anxiety. The scores range from 20 to 80. Reversed scoring items are 1, 2, 5, 8, 10, 11, 15, 16, 19, and 20.

Statement		Not at all	Somewhat	Moderately so	Very much so
1	I feel calm	1	2	3	4
2	I feel secure	1	2	3	4
3	I am tense	1	2	3	4
4	I feel strained	1	2	3	4
5	I feel at ease	1	2	3	4
6	I feel upset	1	2	3	4
7	I am presently worrying over possible misfortunes	1	2	3	4
8	I feel satisfied	1	2	3	4
9	I feel tightened	1	2	3	4
10	I feel comfortable	1	2	3	4
11	I feel self-confident	1	2	3	4
12	I feel nervous	1	2	3	4
13	I am jittery	1	2	3	4
14	I feel indecisive	1	2	3	4
15	I am relaxed	1	2	3	4
16	I feel content	1	2	3	4
17	I feel worried	1	2	3	4
18	I feel confused	1	2	3	4
19	I feel steady	1	2	3	4
20	I feel pleasant	1	2	3	4

**Table 2:** ** RSS.** Ramsay sedation scale (RSS) was used to assess the degree of sedation. The RSS consists of six levels, each describing a different level of sedation.

SCORE	
1	Anxious and agitated or restless or both
2	Co-operative, oriented and tranquil
3	Responding to commands only
4	Brisk response to light glabellar tap or loud auditory stimulus
5	Sluggish response to light glabellar tap or loud auditory stimulus
6	No response to stimulus

**Table 3:** ** Demographic data.** Patient characteristics, vital signs, initial RSS, or STAI-S scores did not differ among the three groups. *Ht: height; Wt: weight;ASA: American Society of Anesthesiologists physical status classification; MBP: mean blood pressure; HR: heart rate; SpO_2_: oxygen saturation; RSS: Ramsay Sedation Scale; STAI-S: State-Trait Anxiety Inventory-State

	Group 1 (n=20)	Group 2 (n=20)	Group 3 (n=20)	P value
Age (years)	73.2 (6.9)	73.6 (4.9)	71.6 (4.5)	.469
Gender (F/M)	16/4	16/4	16/4	1.000
Ht (cm)	156.2 (8.4)	154.6 (5.0)	156.1 (8.6)	.762
Wt (kg)	65.8 (10.1)	62.9 (7.8)	61.7 (8.7)	.348
ASA (I/II)	0/20	1/19	0/20	1.000
MBP (mmHg)	98.9 (8.9)	104.7 (15.9)	104.6 (15.0)	.308
HR (bpm)	63.5 (10.6)	69.0 (10.0)	66.3 (12.9)	.303
SaO_2_ (%)	96.7 (1.5)	96.9 (1.9)	96.7 (1.1)	.912
RSS initial	1.0 (1.0, 1.0)	1.0 (1.0, 1.0)	1.0 (1.0, 1.0)	.158
STAI-S initial	45.7 (16.6)	52.9 (13.4)	55.7 (10.3)	.066

**Table 4:** ** Descriptive statistics.** To compare the power of bandwidth (BW) and anxiety levels (STAI-S), all data from the 60 patients were merged and analyzed. *Ht, height; Wt, weight; MBP, mean blood pressure; HR, heart rate; SpO_2_, oxygen saturation; RSS, Ramsay Sedation Scale; STAI-S, State-Trait

N =180	Minimum	Maximum	Mean (SD)
Age (years)	65	89	72.8 (5.5)
Ht (cm)	141.0	176.0	155.6 (7.4)
Wt (kg)	50.0	82.0	63.5 (8.9)
MBP (mmHg)	56	138	93.3 (16.0)
HR (bpm)	35	96	63.2 (11.0)
SaO_2_ (%)	94	100	97.8 (1.6)
RSS	1	6	1.87 (1.59)
STAI-S	20	76	48.1 (14.9)
BW 1	0.17	0.93	0.49 (0.16)
BW 2	0.31	0.98	0.69 (0.15)
BW 3	0.34	0.95	0.70 (0.14)
BW 4	0.34.	0.99	0.67 (0.14)
